# One hundred fifty years of total laryngectomies

**DOI:** 10.3389/fonc.2024.1351549

**Published:** 2024-06-10

**Authors:** Stéphane Hans, Robin Baudouin, Marta P. Circiu, Florent Couineau, Tiffany Rigal, Marc J. Remacle, Andrea De Vito, Giovanni Cammaroto, Lise Crevier-Buchman, Jérôme R. Lechien

**Affiliations:** ^1^ Research Committee of the Young Otolaryngologists of the International Federation of Oto-rhino-laryngological Societies (YO-IFOS), Paris, France; ^2^ Department of Otorhinolaryngology and Head and Neck Surgery, Foch Hospital, School of Medicine, Simone Veil, Université Versailles Saint-Quentin-en-Yvelines (Paris Saclay University), Paris, France; ^3^ Department of Otolaryngology-Head and Neck Surgery, Forlì & Faenza Hospitals Ravenna & Lugo Hospitals, Health Local Agency of Romagna, Forli, Italy; ^4^ Department of Human Anatomy and Experimental Oncology, Faculty of Medicine, University of Mons (UMONS) Research Institute for Health Sciences and Technology, University of Mons (UMons), Mons, Belgium

**Keywords:** total laryngectomy, history, head and neck surgery, cancer, larynx, otolaryngology, FORLI

## Abstract

The inaugural total laryngectomy in history was conducted by Billroth in 1873. Nevertheless, significant enhancements to the technique were achieved through the contributions of Gluck, Sorensen, and various other surgeons. Throughout the twentieth century, advancements in anesthesia, infectious disease, hospital hygiene, antibiotic therapy, resuscitation, and the expertise of numerous laryngologists elevated total laryngectomy to a pivotal surgical intervention in head and neck surgery. The latter half of the twentieth century witnessed a paradigm shift with the emergence of organ preservation protocols. Total laryngectomy became the preferred choice for patients experiencing radiotherapy failure. However, the widespread use of laryngeal conservative treatments appears to be correlated with a decline in overall survival rates in the United States and Europe. The evolution of new minimally invasive surgical approaches in the twenty-first century may usher in a revolutionary era in the management of laryngeal carcinoma, offering the potential for improved survival and functional outcomes.

## 1 Introduction

The year 2023 marks the 150th anniversary of the inaugural total laryngectomy (TL) performed for laryngeal cancer by the Austrian surgeon Theodor Billroth (1). TL is a common surgical procedure in contemporary head and neck oncology, particularly for primary advanced or recurrent laryngeal cancer, which concerns approximately 200,000 new cases globally each year (2). In this historic paper, we delineated the evolution of the TL technique and described the influence of organ preservation protocols in the management of advanced laryngeal cancers.

## 2 The pioneers

The concept of laryngeal excision for malignancy emerged in the first half of the nineteenth century. However, numerous obstacles surrounded TL at that time, including the necessity for general anesthesia, blood transfusion, and antibiotic therapy. Johann Friedrich Hermann Albers (1805–1867) was the first surgeon to perform TL on beagle dogs in Bonn (Germany) in 1829 (3). Among the experiments, one dog remarkably survived for 9 days post-procedure. Dr. Albers subsequently published the inaugural monograph on diseases of the larynx (4). A few decades after the Albers’ works, German physician Bernhard Rudolf Konrad von Langenbeck (1810–1887) proposed the realization of TL in human patients in 1854. As a professor of surgery in Germany, Dr. von Langenbeck taught some surgeons, including Theodor Billroth (1829–1894) and, later, Themistocles Gluck (1853–1942) (5). Contrary to some literature, the first TL was not carried out by the British surgeon Patrick H. Watson (1832–1907) in 1866. An examination of historic medical records revealed that Watson only performed an “*in vivo*” tracheotomy on a patient with syphilitic laryngitis, while the TL was performed post-mortem for educational and demonstration purposes (6).

## 3 The first total laryngectomy in human

The inaugural TL was carried out by Billroth on 31 December 1873 for a laryngeal cancer. In 1870, Billroth’s assistant, Vincenz Czerny (1842–1916), had experimentally outlined the procedure on dogs, yielding controversial results with an 80% mortality rate, which led to the discontinuation of the experiments (1). Three years later, Billroth performed the first human TL on New Year’s Eve for a 36-year-old individual. Having previously attempted a median cricothyroidotomy and endolaryngeal excision of the tumor without success, the unplanned TL intervention was carried out due to the patient’s deteriorating condition. Described as a surgery marked by bleeding, intermittent awakening from anesthesia, and cough, the procedure resulted in a postoperative pharyngocutaneous fistula, yet the patient successfully resumed oral feeding. In the subsequent months, the patient used an artificial larynx crafted by Carl Gussenbauer (1842–1903). Unfortunately, the patient survived for only 7 months (7). The success of this initial TL did not prompt widespread adoption of the technique, as nearly half of the patients succumbed to complications such as fistulas, hemorrhage, shock, mediastinitis, and bronchopneumonia (8, 9). At the first International Laryngological Conference in London (1881), most of the head and neck surgeons discouraged the practice of TL due to the low survival outcomes (10). According to the literature, the first long-term survivor after TL was a patient with a laryngeal sarcoma operated on by Enrico Bottini (1835–1903) in 1875, who survived during 15 years (11).

## 4 The works of Gluck and Sorensen

In 1888, John Sendziak reported data from 110 TL, revealing a mortality rate of 44.7% (12). Between 1889 and 1900, the postoperative mortality rate decreased from 44% to 8.5%, and the 3-year overall survival rate improved from 44% to 58% (13). These positive changes were attributed to technical modifications implemented by Themistocles Gluck and Johannes Sörensen. In 1877, Gluck served as an army general surgeon during the Russo–Turkish War (1877–1878), an experience that contributed to the development of an important aspect of the laryngectomy concept: the “prophylactic resection of the trachea.” This concept emerged after treating a patient with a gunshot laryngeal wound. A publication from 1881 by Gluck and Zeller detailed the separation of the trachea from the larynx and the dissection of the pharyngoesophageal space. The trachea’s orifice was sutured directly to the skin of the neck, establishing the current concept of definitive tracheostomy. Over time, Gluck and Sörensen revised the technique, reaching excellent results (3). The separation of the airway from the digestive tract (neopharynx) significantly reduced the risk of inhalation pneumonia.

In the 1890s, French surgeons Jules Péan (1830–1898), Louis Ollier (1830–1900), and Constant Vanlair (1839–1914) embraced Gluck’s concept of prophylactic tracheal resection. In the early part of the nineteenth century, the recognition of the necessity to separate the trachea from the pharyngoesophagus extended to Francesco Durante (1844–1934) in Italy (14) and Jacob da Silva Solis-Cohen (1838–1927) in the USA (15).

Themistocles Gluck’s contributions gained official acknowledgment in the early twentieth century, particularly when he was nominated for the Nobel Prize in Physiology or Medicine in 1933 (16). TL was indicated in advanced laryngeal cancer, which was a challenging diagnosis given the high prevalence of laryngeal tuberculosis or syphilis. The complexities associated with differential diagnoses significantly reduced with the discovery of *Mycobacterium tuberculosis* in 1882 by Robert Koch (1843–1910) and the introduction of syphilis testing (Bordet–Wassermann reaction) in 1906 by Jules Bordet (1870–1961) and August von Wassermann (1866–1925). Then, the improvement of TL indications, procedures, and complications coincided with substantial advancements in diagnosis during the same period (17).

## 5 The end of the nineteenth century and the first part of the twentieth century

To the end of the nineteenth century, two primary techniques for TL were used. The first, reported by Charles Périer in 1890, contrasted with the second technique described by Gluck and Sorensen in 1895 (18–20). Both differed in the surgical planning, with one adopting a top–down approach and the other a bottom–up approach.

During this era, high rates of postoperative complications and inappropriate indications resulted in disappointing outcomes. In that way, some practitioners more frequently advocated for a palliative tracheostomy instead of TL. In 1897, Sendziak reported a mortality rate of 44.7% and a 3-year overall survival of 5.85%, reflecting the challenging landscape of the procedure (19).

The discovery of cocaine, procaine, and adrenaline facilitated the advancement of local anesthetic techniques and improvements in operating room conditions. Simultaneously, the procedures developed by Périer and Gluck reached significant enhancements in tissue incisions, operating times, subhyoid muscle management, two-plane pharyngeal sutures (mucous and muscle), skin sutures, and the tracheal stoma.

At this time, the number of TL steps was a controversial point. In France, Georges Portmann (1890–1985) and colleagues proposed a three-step procedure, encompassing 1) tracheostomy, 2) laryngectomy with pharyngostomy, and 3) closure of the pharyngostomy 2 months after the TL. They reported a 100% immediate postoperative success rate in a cohort of 51 patients in 1937, emphasizing the impact of their approach on postoperative mortality (10, 21–23).

Based on the works of Périer and Gluck, various procedures were proposed, including the three-step TL by Jean Leroux-Robert (1907–1998). Leroux-Robert’s approach involved tracheotomy, pharyngolaryngeal detachment, and laryngectomy, contributing to the evolving landscape of TL procedures (19, 21–23).

## 6 The second part of the twentieth century

Advancements in anesthesiology, hygiene, and infectious disease continued to influence the TL technique and outcomes. Post-World War II, TL procedures evolved to be performed under general anesthesia with intubation, using a one-step approach with a top–down or bottom–up direction based on tumor location and surgical team preference. The timing of tracheotomy, initially conducted under local anesthesia at the surgery’s commencement, was strategically postponed to the most opportune moment to minimize unnecessary detachments. The steps of TL were influenced by the post-World War II era and the related introduction of the first anesthesia ventilators (24). Moreover, in the second part of the twentieth century, there was a growing emphasis on respiratory function, voice rehabilitation (25), and overall quality of life (26, 27). Consequently, an increasing number of publications focused on post-TL voice rehabilitation, partial laryngectomies (28), and organ preservation treatments for advanced laryngeal cancer (29, 30). Despite the emergence of radiation therapy, TL remained common due to cost-effective considerations compared to organ preservation approaches (31). The development of neck dissection techniques within the twentieth century improved survival outcomes associated with TL. George Washington Crile (1864–1943) proposed the first standardized neck dissection procedure in 1906 (32). Over subsequent decades, various head and neck surgeons, including Martin, Bocca, Lindenberg, Guerrier, Shah, and Medina, modified lymph node dissection procedures, contributing to the advancement of overall survival in TL (17, 18, 21).

## 7 Organ preservation strategies

In the twentieth century, radiotherapy emerged as an alternative treatment for laryngeal carcinoma (33). Due to anatomical defect and mutilation associated with TL, radiation became the primary treatment in many hospitals, despite the potential risk of failure and the need for salvage TL. In 1991, the “VA protocol” (28) revealed a predictive value of a patient’s response to induction chemotherapy on their response to laryngeal radiation. Patients with a positive response to the induction chemotherapy were treated with radiation, while TL was proposed for non-responders. This protocol was associated with a laryngeal preservation in a substantial number of patients with advanced cancers, reporting comparable local control and overall survival to TL patients.

Another significant therapeutic advancement was achieved through the RTOG 91–11 study (29), which compared three groups of patients: 1) patients with advanced laryngeal carcinoma treated with radiotherapy, 2) patients treated with concurrent chemoradiotherapy (CRT) using cisplatin, and 3) patients undergoing two cycles of induction chemotherapy (cisplatin and 5-fluorouracil) followed by a third cycle and radiotherapy for responders or TL for non-responders. The results indicated similar overall survival among the groups, with superior local control in the CRT group compared to the others (29). In the RTOG 91–11 study, the cumulative 10-year grade III–V toxicity rate with concurrent CRT was 33%, encompassing mucosal atrophy, fibrosis, induration, skin ulceration, xerostomia, dysphagia, neuropathies, pain, pneumonitis, and cardiomyopathy. Approximately one-fifth of patients with advanced (UICC stage III/IV) glottic or supraglottic laryngeal carcinoma required TL for residual or recurrent tumor within the 10-year follow-up post-CRT (34).

In the early twenty-first century, Bonner et al. reported that treating locoregionally advanced head and neck carcinoma with concomitant high-dose radiotherapy plus cetuximab improved locoregional control and reduced mortality without increasing the common toxic effects associated with head and neck radiotherapy (35). However, the chemotherapy induction trial is not an option for patients with significant comorbidities (36, 37). Nowadays, treatment decisions are made through a multidisciplinary oncological board, which may indicate several strategies discussed with the patients.

## 8 Evolution and current survival outcomes after total laryngectomy versus conservative treatments

The survival after surgical treatment for laryngeal cancer is influenced by comorbidities, cTNM stage, and the localization of the primary tumor. The 5-year overall survival for laryngeal squamous cell carcinoma is 60%. Among TL patients, the lowest overall survival is observed in subglottic (40%) and retrocricoid tumors (15%), while the highest overall survival is shown in patients with glottic cancer (70%), which is attributed to early symptoms and related detection (38). Approximately 30% of patients experience locoregional and distant recurrences or develop second primary cancer within a year post-TL (39). A closed follow-up and the realization of imaging are important in enhancing the early detection of recurrence or second primary cancer. The overall survival of individuals with salvage laryngectomy is lower compared to those successfully treated with radiation, with a 5-year overall survival rate of 37% for stage III/IV laryngeal cancer (40).

To date, cT3 laryngeal tumors are primarily managed with CRT, offering TL as an option in the case of conservative treatment failure. However, radical surgery may still be indicated in cases where there is destruction in the laryngeal skeleton (cT4a). While CRT can spare the need for TL, it may result in a dysfunctional larynx and, in cases of recurrence, challenges in confirming the cancer diagnosis. Additionally, TL patients who undergo conservative CRT may experience a higher occurrence of postoperative complications compared to those undergoing primary TL. An examination of the National Cancer Data Base (NCDB) revealed 158,426 cases of laryngeal squamous cell carcinoma diagnosed between 1985 and 2001, confirming a noted trend toward decreasing survival in laryngeal cancer patients. The decreased survival observed in the mid-1990s may be linked to changes in management patterns, with an increase in CRT and a decline in primary surgery. A European study (41) documented a reduction in surgery as the initial treatment and a shift from induction chemotherapy to CRT as an organ preservation strategy. Patients treated in the last decade (2005–2014) exhibited worse cancer-specific survival compared to those treated in the previous decade (1995–2004).

## 9 Salvage total laryngectomy following organ preservation strategies

Laryngeal cancer recurrence after non-surgical treatment commonly requires salvage surgery such as salvage laryngectomy. The 5-year overall survival rate after salvage laryngectomy is approximately 50%. Prognostic factors include advanced recurrent stage, severe medical comorbidities, and recurrent adenopathy findings. In the salvage setting, compared to primary surgery, surgical complications are heightened with the most prevalent complication being pharyngocutaneous fistula (PCF).

De Virgilio et al. reported on 1,694 patients that pedicled flaps significantly reduced the PCF rate (OR: 0.35, CI: 0.20–0.61) (42). Salivary bypass tubes (SBT) are increasingly used to prevent pharyngocutaneous fistula (PCF) following laryngectomy and pharyngolaryngectomy. A recent study on 1,960 patients with SBT suggested a significant reduction in the incidence of PCF and pharyngeal stenosis (PS) after TL (43). The salvage TL necessitates protecting the neck vessels with a flap, and the use of a salivary bypass tube may be considered.

## 10 Minimal invasive transoral total laryngectomy

In 2022, we published two articles detailing the historical evolution of surgical treatment for laryngeal cancers (28, 44). Over the past three decades, there has been a significant transformation in the surgical approach to laryngeal cancer, transitioning from external surgery to minimally invasive techniques such as laser and robotic surgery (28, 44). In the last decade, transoral robotic surgery (TORS) has progressed from a proof-of-concept to becoming a standard-of-care approach in hospitals with high-volume robotic surgeries. However, the current robotic system is not universally suited for all types of head and neck surgeries. Currently, accepted indications for TORS are primarily cT1–T2, with some selected cT3–T4a cases in the oropharynx and supraglottic regions where the surgeon can achieve adequate instrument-optic view triangulation.

The first reported instance of transoral robotic surgery total laryngectomy (TORS-TL) was documented in 2013 through a French–American cadaver study and case series using the da Vinci System (45). At that time, authors suggested that this approach was particularly suitable for patients undergoing salvage laryngectomy after radiation failure. Since then, only four small cohort studies have been published, yielding controversial findings (46–50).

While TORS TL appears to be safe and effective, its exact indications and limitations remain undefined. Key advantages of TORS-TL include the absence of scarring outside the tracheostomy and the avoidance of flaps to protect the vessels. However, notable disadvantages include prolonged procedure times and associated costs.

A Spanish team has developed a minimally invasive, transoral, endoscopic, and non-robotic approach utilizing transoral ultrasound surgery (TOUSS) for laryngeal and pharyngeal tumors (51). In 2016, the same team reported two cases of TL using this ultrasonic scalpel-based technique, with total surgical times of 210 and 180 min, respectively (52). The advantages of TOUSS include the absence of neck scars, musculocutaneous flaps, and a reduced pharyngotomy size, leading to decreased morbidity for the patient without the added costs associated with TORS. Currently, the adoption of these two techniques, TORS-TL or TOUSS-TL, is limited.

## 11 Conclusion

The first historical TL was performed by the Austrian surgeon Theodor Billroth in 1873, in an emergent context. However, the technique underwent significant refinement through the contributions of Gluck, Sorensen, and numerous other surgeons. Throughout the twentieth century, advancements in anesthesia, infectiology, hospital hygiene, antibiotic therapy, resuscitation, and the expertise of laryngologists established TL as a pivotal surgical intervention in head and neck surgery. A paradigm shift occurred in the twentieth century with the introduction of organ preservation protocols. TL became the preferred approach in cases of radiotherapy failure. However, the widespread use of laryngeal conservative treatments appears to be linked to a decrease in overall survival rates in both the United States and Europe. The development of new minimally invasive surgical approaches in the twenty-first century holds the potential to usher in a new era in the management of laryngeal carcinoma, offering improved survival and functional outcomes.

## Author contributions

SH: Writing – original draft. RB: Writing – review & editing. MC: Writing – review & editing. FC: Writing – review & editing. TR: Writing – review & editing. MR: Writing – review & editing. ADV: Writing – review & editing. GC: Writing – review & editing. LC-B: Writing – review & editing. JL: Writing – review & editing.
